# Anabolic–androgenic steroids: How do they work and what are the risks?

**DOI:** 10.3389/fendo.2022.1059473

**Published:** 2022-12-19

**Authors:** Peter Bond, Diederik L. Smit, Willem de Ronde

**Affiliations:** ^1^ PeterBond.org, Zeist, Netherlands; ^2^ Department of Internal Medicine, Elisabeth-TweeSteden Hospital, Tilburg, Netherlands; ^3^ Department of Internal Medicine, Spaarne Gasthuis, Haarlem, Netherlands

**Keywords:** androgens, androgen abuse, anabolic steroids, bodybuilding, doping in sports

## Abstract

Anabolic–androgenic steroids (AAS) are a class of hormones that are widely abused for their muscle-building and strength-increasing properties in high, nontherapeutic, dosages. This review provides an up-to-date and comprehensive overview on how these hormones work and what side effects they might elicit. We discuss how AAS are absorbed into the circulation after intramuscular injection or oral ingestion and how they are subsequently transported to the tissues, where they will move into the extravascular compartment and diffuse into their target cells. Inside these cells, AAS can biotransform into different metabolites or bind to their cognate receptor: the androgen receptor. AAS and their metabolites can cause side effects such as acne vulgaris, hypertension, hepatotoxicity, dyslipidemia, testosterone deficiency, erectile dysfunction, gynecomastia, and cardiomyopathy. Where applicable, we mention treatment options and self-medication practices of AAS users to counteract these side effects. Clinicians may use this review as a guide for understanding how AAS use can impact health and to assist in patient education and, in some cases, the management of side effects.

## 1 Introduction

**Figure 1:**
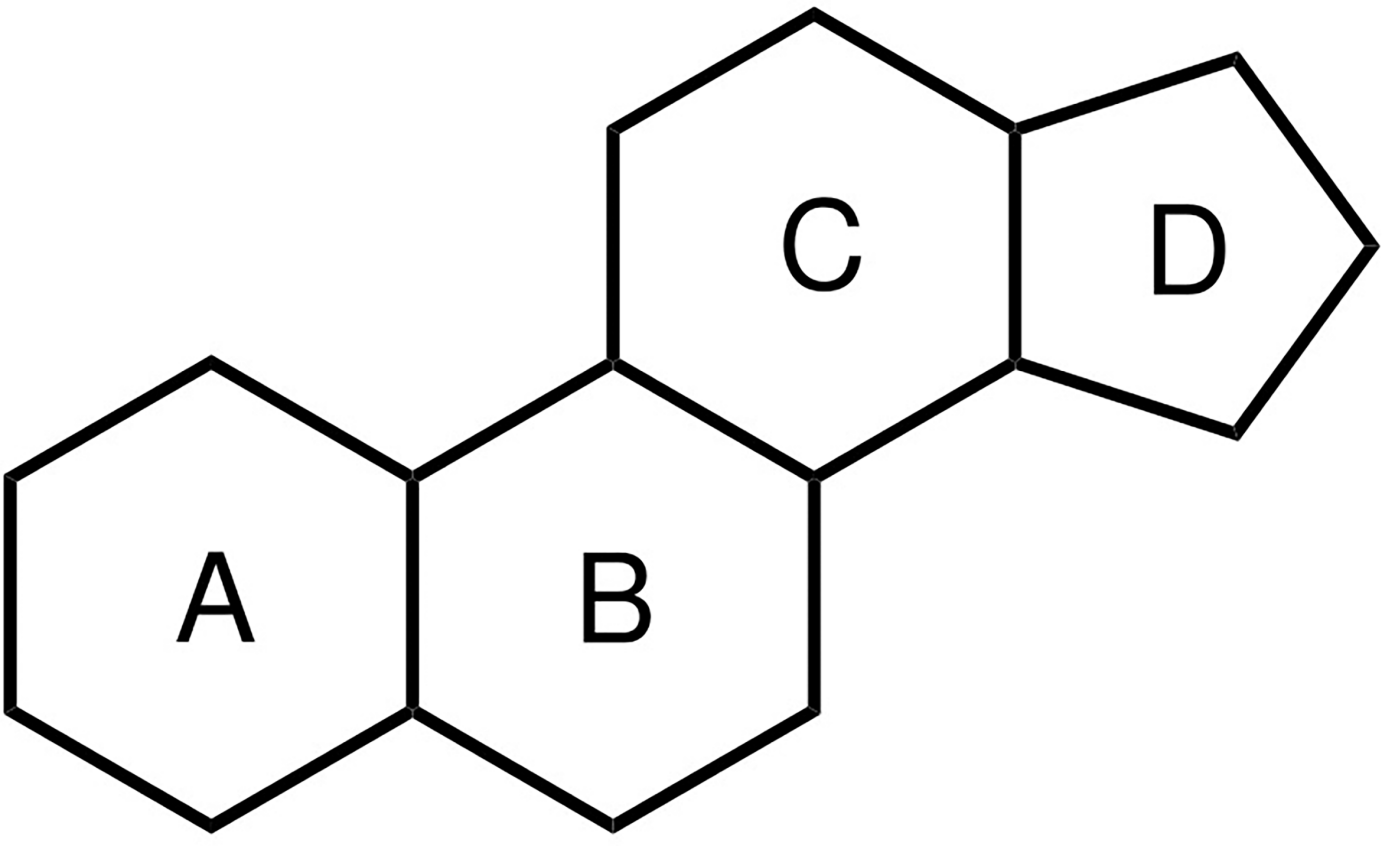
Chemical structure of the steroid nucleus consisting of three cyclohexane rings **(A–C)** and one cyclopentane ring **(D)**.

Anabolic–androgenic steroids (AAS) are a class of natural and synthetic hormones that owe their name to their chemical structure (the steroid nucleus, see **Figure 1**) and the biological effects (anabolic and androgenic) they induce. Anabolic refers to the skeletal muscle-building properties of AAS, whereas androgenic refers to the induction and maintenance of male secondary sexual characteristics (which in principle includes the anabolic action, thereby rendering the term an oxymoron (1)). Testosterone is the primary endogenous hormone belonging to this class. It is widely used therapeutically, in various esterified forms, as replacement therapy in male hypogonadism. Testosterone, and a select few other AAS such as nandrolone and oxandrolone, might also be prescribed for other medical conditions (e.g., osteoporosis or aplastic anemia). Besides this valid medical use, AAS are widely used – or rather, *abused* – for their muscle-building and strength-increasing properties in dosages far exceeding those used therapeutically. For brevity, in the remainder of this review we employ the term ‘AAS use’ to refer to the nonmedical high-dose abuse of AAS.

AAS are easily acquired through local dealers or the internet, even though their trade, and sometimes also their use, is illegal in many countries. With an estimated global lifetime prevalence rate of 3.3% (6.4% for males and 1.6% for females) (2), virtually every practising physician will provide care for an AAS user at some point in their career. Although, of course, the AAS user will not necessarily disclose his use of AAS or present with side effects caused by it. Given the health risks involved with AAS use, as well as the high lifetime prevalence, it is prudent for physicians, in particular general practitioners, endocrinologists, sports medicine, and addiction medicine physicians, to be knowledgeable about AAS use and its possible adverse health effects to provide appropriate care and establish rapport with this group of patients. This review therefore provides a comprehensive overview of this class of hormones’ basic pharmacology and side effects. Throughout this review, we mention treatment options for several side effects; these should not be considered strict recommendations, as they are largely a reflection of how AAS users self-medicate or what is known from the literature. It remains debatable whether or not physicians should medically target unwanted effects of AAS use. Obviously, discontinuing AAS would be the preferred solution for virtually every side effect. This could be encouraged by patient education on the possible risks and addressing psychological issues that maintain AAS use, such as body dysmorphia and addiction. If the patient is nonetheless unwilling to quit, physicians may consider treatment to reduce harm, as is customary in the approach and treatment of, for example, smokers and alcoholics.

## 2 From administration to action

AAS are most commonly administered by intramuscular (i.m.) injection or by oral ingestion. AAS formulations for i.m. injection are based on vegetable oils, such as arachis oil, in which AAS are dissolved. Aromatic compounds such as benzoyl benzoate (BB) or benzyl alcohol (BA) are often added as excipients for their bacteriostatic properties and to increase the oil solubility of AAS. After injection, an oil depot forms inside the muscle tissue and spreads along the muscle fibers – seemingly squeezed between them – forming an elongated shape (3). The AAS gradually diffuse out of the oil depot and into the interstitial fluid. The rate at which this occurs strongly depends on the carboxylic acid group that is attached onto the parent molecule at carbon 17 of the steroid nucleus. This attachment – esterification of the 17*β*-hydroxyl group – greatly retards the release of the compound from the oil depot by increasing its partition coefficient, i.e., making it more lipophilic and less hydrophilic. For example, unmodified testosterone reportedly has a half-life of approximately 10 minutes when injected (4), whereas 17*β*-OH esterification by a 3-carbon carboxylic acid group (propionate) or 7-carbon carboxylic acid group (enanthate) prolongs its half-life to approximately 1.0 (4) and 4.2 days (5), respectively. The injection site and volume may also affect pharmacokinetics (6). Once the esterified steroid molecule reaches the systemic circulation, either *via* direct diffusion or lymphatic drainage of the interstitial fluid, esterases cleave off the ester group, releasing the parent compound (7).

Orally ingested AAS are rapidly absorbed in the gastrointestinal (GI) tract, with serum concentrations peaking 1–2 hours after ingestion of methyltestosterone (8). The absorbed AAS reach the liver *via* the portal vein. Without structural modification to resist first-pass metabolism, a large fraction of the absorbed AAS will be metabolized before leaving the liver and re-entering the circulation. This drastically decreases oral bioavailability. For example, after oral administration of 25 mg testosterone, less than 1 mg (4%) becomes systemically available (9). The oral bioavailability of AAS can be increased by making the parent molecule more lipid-soluble by the esterification process described in the previous paragraph. This modification allows a larger fraction of the absorbed AAS to enter the lymphatic system and bypass first-pass metabolism. However, even after esterification of testosterone by an 11-carbon carboxylic acid group (undecanoate), oral bioavailability remains poor at 6.8% (9). The formulation also shows large intra- and interindividual variation in bioavailability, making it cumbersome for replacement therapy. To address this, testosterone undecanoate has recently been formulated in a self-emulsifying drug delivery system (SEDDS) to further enhance lymphatic absorption and reduce intra- and interindividual variation (10). The FDA approved such a formulation (brand name JATENZO^®^) in 2019. However, it needs to be dosed twice daily to maintain physiological testosterone levels throughout the day. An alternative strategy centers around significantly retarding metabolism of the compound. This can be achieved by 17*α*-methylation of the parent molecule. This structural modification greatly increases oral bioavailability, although it comes with hepatotoxicity (11).

**Figure 2:**
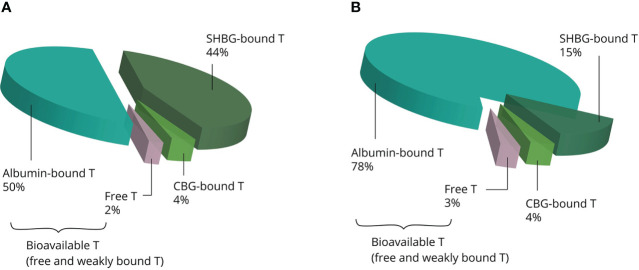
Examples of the estimated distribution of testosterone bound to binding proteins at physiological concentrations **(A)** and supraphysiological concentrations **(B)**. The albumin-bound and unbound fractions become larger, whereas the SHBG-bound fraction becomes smaller with increasing concentrations. Abbreviations: SHBG, sex hormone-binding globulin; CBG, corticosteroid-binding globulin; T, testosterone.

Once in the systemic circulation, AAS are transported to the tissues bound to binding proteins: albumin, sex hormone-binding globulin (SHBG), corticosteroid-binding globulin (CBG) and orosomucoid. Under physiological conditions, testosterone is predominately bound to the first two, leaving only 1% to 4% of circulating testosterone unbound (12). SHBG binds testosterone with high affinity but has a relatively low binding capacity. Conversely, albumin binds testosterone with low affinity but has a virtually limitless binding capacity (13). SHBG is present in the bloodstream as a homodimer, with each monomer having one steroid-binding site (14). Under physiological conditions about one third of the binding sites is occupied by testosterone, with further occupation by several other steroids rendering about 44% of SHBG-binding sites unbound (13). With physiological SHBG levels in the 10–56 nmol/L range, it is clear that supraphysiological dosages of testosterone saturate its binding capacity. Besides, supraphysiological dosages strongly decrease circulating SHBG levels (15). Thus, increasing dosages of testosterone result in a larger fraction of albumin-bound testosterone relative to the SHBG-bound fraction (see **Figure 2**). It is noteworthy that SHBG has very low affinity for other commonly used AAS, such as nandrolone, methenolone, stanozolol, methandienone, fluoxymesterone and oxymetholone (16).

While not solely responsible for all of testosterone’s effects, the unbound fraction is suggested to be a more accurate reflection of systemic androgenic action than total testosterone. Low free testosterone levels are associated with androgen deficiency-related symptoms in the presence of normal total testosterone levels, while normal free testosterone levels are not associated with androgen deficiency-related symptoms in the presence of low total testosterone levels (17). Regardless, the relative importance of the unbound and bound fractions is still a matter of debate, as current evidence simply falls short from settling the matter (18).

**Figure 3:**
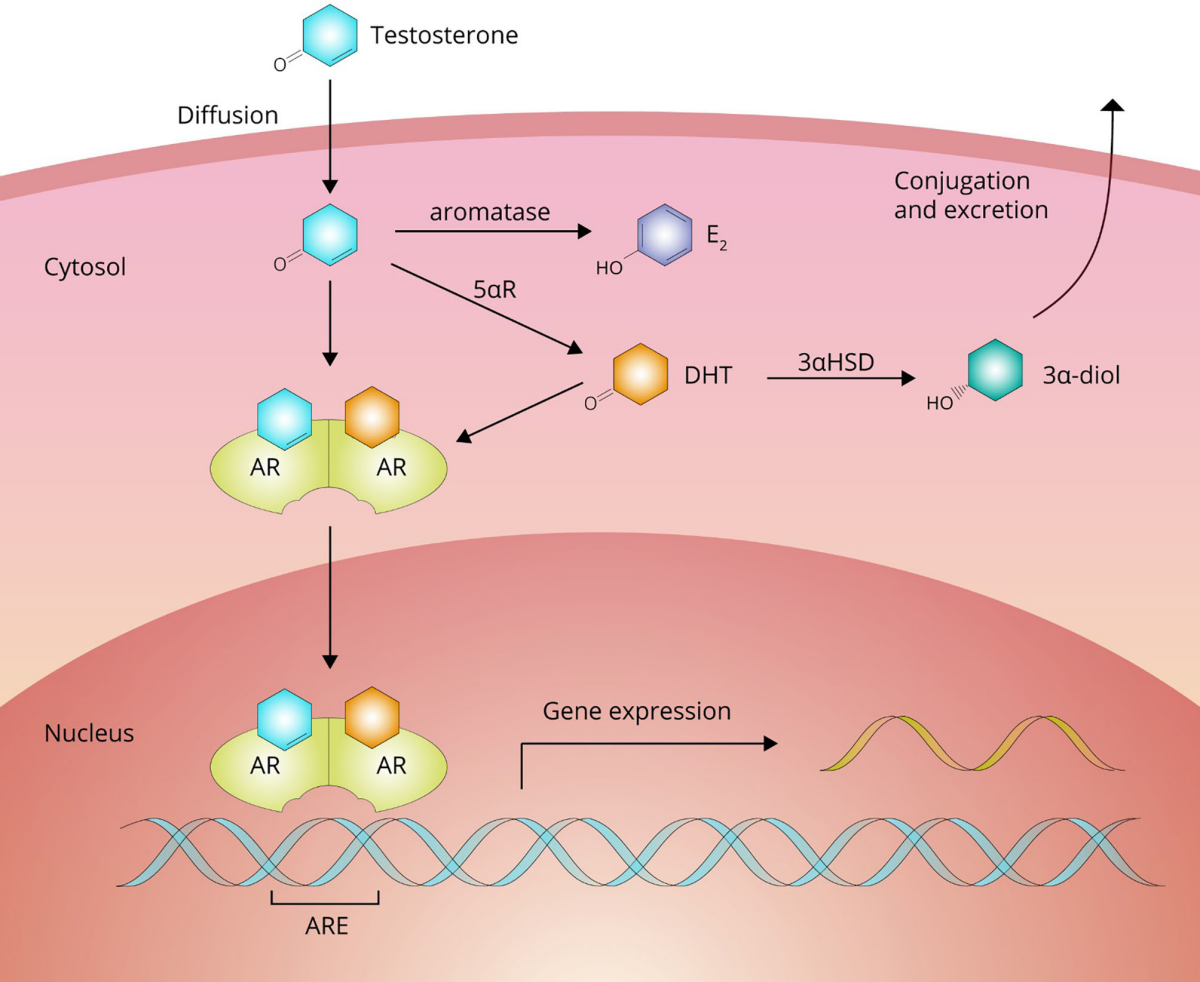
Testosterone passes the cell membrane by passive diffusion. Inside the cell, it can either bind directly to the androgen receptor (AR) to affect gene expression or undergo bioactivation into dihydrotestosterone (DHT) by 5*α*R-reductase (5*α*R) family enzymes or estradiol (E_2_) by aromatase. DHT can be subsequently inactivated to 3*α*-androstanediol (3*α*-diol) by 3*α*-hydroxysteroid-dehydrogenase (3*α*HSD).

From the bloodstream, AAS move into the extravascular compartment and diffuse to their target cells to exert their effects. After crossing the plasma membrane, AAS can undergo biotransformation or bind to their cognate receptor, the androgen receptor (AR; see **Figure 3**). Biotransformation can occur in three directions: 1) into a more potent androgen, 2) into a less potent or inactive metabolite, and 3) into an estrogen.

Testosterone is bioactivated into a more potent androgen in tissues expressing enzymes of the 5*α*-reductase family. These enzymes catalyze a reduction reaction at carbon 5, adding an *α*-oriented hydrogen atom. With testosterone as a substrate, this reaction yields the most potent naturally occurring androgen, namely, dihydrotestosterone (DHT). In adults these enzymes are expressed, among other tissues, in the liver, skin, prostate, epididymis, seminal vesicles, testis, kidney, pancreas and brain (19). It should be noted that DHT is not thought to contribute to the muscle-building effects of testosterone. DHT levels are (very) low in skeletal muscle as it does not significantly express the enzyme. DHT also appears to be broken down in skeletal muscle by inactivation to 3*α*-androstanediol by the enzyme 3*α*-hydroxysteroid-dehydrogenase (20, 21). Indeed, changes in fat-free mass in response to graded doses of testosterone are unaffected by DHT suppression with the potent 5*α*-reductase inhibitor dutasteride (22). The conversion of testosterone to DHT shows saturable Michaelis-Menten kinetics with an estimated *in vivo* K_m_ value of 3.35 nM (23). Bioactivation through this pathway into a more potent androgen does not appear to occur for any of the other commonly used AAS (24).

AAS are predominantly bioinactivated in the liver, but also in the kidneys and various other androgen-sensitive tissues (25). AAS are subject to both phase I and phase II metabolism. In general, phase I metabolism mainly involves reduction at carbons 3 and 5 of the A-ring and oxidation of the hydroxyl group at carbon 17 of the D-ring of the steroid nucleus (24). These phase I metabolites might then undergo conjugation into glucuronides and be subsequently excreted (26). Some sulfation also occurs (27).

Bioactivation into an estrogen can occur with AAS that are a substrate for the aromatase enzyme. This pathway is particularly relevant for testosterone (yielding 17*β*-estradiol). Most other AAS are not a substrate for aromatase or are converted at lower rates, although the latter group can still yield a considerable amount of estrogen if administered in high doses. The produced estrogen subsequently exerts its effects by binding to estrogen receptors *α* and *β*, thereby diversifying the biological effects of the parent compound. Estrogen production is especially relevant in light of the development of gynecomastia and the negative endocrine feedback exerted on the hypothalamic–pituitary–gonadal axis (HPGA). We describe its role herein in further detail in the subsections on Gynecomastia and Testosterone Deficiency.

In its unliganded state, the AR is predominately located in the cytoplasm in association with chaperone proteins (28). Ligand binding initiates a cascade of events that leads to translocation of the AR-ligand complex from the cytoplasm into the nucleus, dissociation of the associated chaperone proteins, and formation of a homodimer that enables binding to the androgen response elements (AREs) found in the regulatory regions of androgen target genes (29). The human genome contains thousands of AR-binding sites (30). Once bound to these sites, the complex regulates gene transcription and thereby exerts its various effects. Additionally, AAS exert nongenomic effects which, at least in part, appear to be mediated by a receptor different from the AR (31, 32). The G-protein coupled receptor GPRC6A is one such candidate (33). The clinical effects that originate from these nongenomic actions are unclear and remain to be characterized.

The overall clinical effects, however, are crystal clear – first and foremost the muscle-building effect pursued by AAS users. In 1996, a milestone randomized-controlled trial by Bhasin et al. demonstrated beyond any doubt that supraphysiological doses of testosterone (up to 600 mg testosterone enanthate weekly) were effective in increasing muscle size and strength in healthy men in a dose-dependent manner, especially when combined with strength training (34). More recent well-designed trials continued to provide further support for the potent muscle-building effects of AAS that had already been recognized by athletes for decades (15, 22, 35–38).

## 3 Side effects

**Figure 4:**
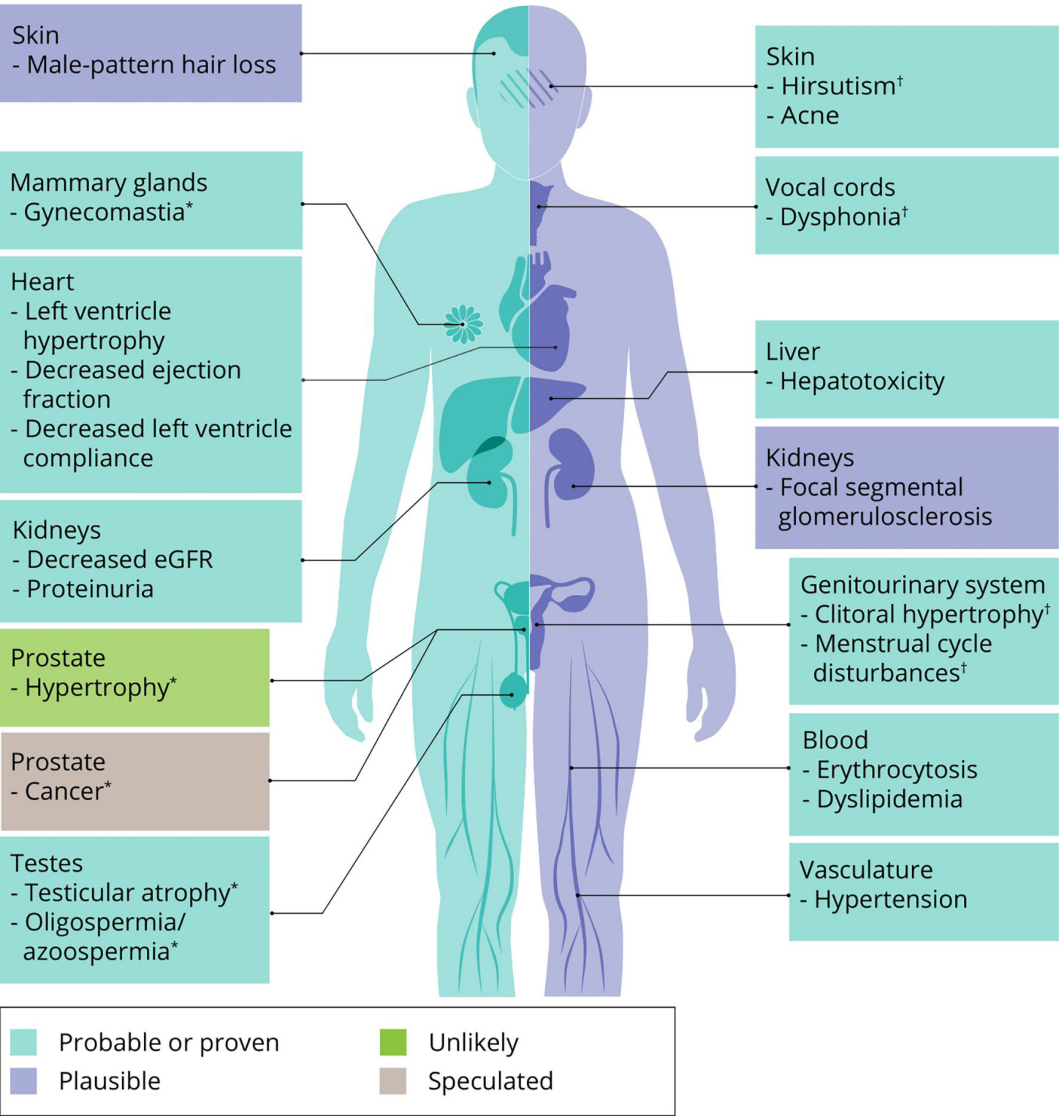
An overview of side effects that can be caused by AAS use. Classification of a side effects’ probability is based on expert opinion of the authors. ∗ specific to men, † specific to women.

The effects of AAS on muscle mass and strength are at the root of this class of drugs’ popularity. However obvious and clear, their use is not without side effects – as also acknowledged by AAS users themselves: all 100 subjects of a recent prospective observational study reported at least one side effect from AAS use (39). What follows is an overview of the most important or frequent side effects of AAS use based on the best available evidence from the literature. Where applicable, we mention treatment options and self-medication practices of AAS users to counteract these side effects. An overview of all side effects covered in this review is illustrated in **Figure 4**.

### 3.1 Erythrocytosis

Erythrocytosis, or polycythemia, an increase in blood hematocrit or hemoglobin levels, is a common side effect of AAS use, even on replacement dosages. It is the most frequent adverse event in older men receiving testosterone replacement therapy (TRT) (40). The underlying mechanism is not completely understood, but evidence points towards the establishment of a new erythropoietin/hemoglobin set point with a concurrent suppression of hepcidin (an inhibitor of gut iron absorption) in response to testosterone administration (41).

The stimulatory effect on erythropoiesis is dose-dependent – at least beyond 300 mg testosterone enanthate weekly – and is more pronounced in older men (42). It takes several months of testosterone treatment before hematocrit stabilizes, with one (uncontrolled) trial reporting a continuous increase in hematocrit up to 12 months in older men receiving testosterone (43). This effect likely does not dependent on 5*α*-reductase. Treating healthy young men with the 5*α*-reductase inhibitors finasteride and dutasteride for one year had no effect on hemoglobin levels (44). Likewise, dutasteride had no effect on hemoglobin levels compared with placebo when used in conjunction with graded doses of testosterone enanthate up to 600 mg weekly (23).

The incidence of erythrocytosis in men on TRT is formulation-dependent. Short-acting i.m. injections of testosterone (cypionate and enanthate esters) are associated with a high incidence of erythrocytosis, whereas pellets, transdermal gels and patches, and long-acting i.m. injections (undecanoate ester) are associated with a low incidence (45). This might be caused by the larger fluctuations in serum concentrations with the use of short-acting i.m. formulations, during which the peaks might cover transient periods of supraphysiological concentrations. Regardless, these findings are unlikely to be of significance in the context of AAS abuse, in which dosages are a multiple of that employed in TRT.

In young men, hemoglobin levels increase by 1.4 g/dL after 20 weeks of 600 mg weekly testosterone enanthate administration (15). This would translate to an approximately 4%-point increase in hematocrit levels. In older men receiving the same dosage for the same duration, hemoglobin levels increase by 2.9 g/dL (37). The HAARLEM study – a large prospective observational study in which users self-administered a mean AAS dosage of 898 mg weekly over a median duration of 13 weeks – showed similar results (46). At the end of their cycle, hematocrit levels had increased by 3%-point compared with baseline. The increase was not associated with longer use or higher AAS dose. A reason for this might be that, beyond a certain level of androgenic activity, no further increase of hematocrit occurs: a ‘ceiling effect’. Notably, whereas only 5% of subjects in this study had hematocrit levels above reference range (*>*50%) at baseline, 33% of subjects had higher than reference levels at the end of their cycle. Hematocrit levels returned to baseline 3 months after the cycle. It is worth noting that a small increase (+46.3·10^9^/L) in platelet count was also observed. This increase correlated positively with the use of oral AAS.

While the clinical implications of an AAS-induced hematocrit increase are unclear, there is reason to believe it might be detrimental to health. The reason for this is that increased blood viscosity is related to thrombosis (47) and hematocrit levels are an important determinant of blood viscosity (48). A large Danish cohort, as part of the Tromsø Study, found a positive association between hematocrit values and venous thromboembolic events (VTE) (49). Their multivariate model, which included age, smoking and BMI as covariates and hematocrit as the continuous variable, found a hazard ratio (HR) of 1.33 (95% CI: 1.05–1.70) for VTE for every 5%-point hematocrit increase in men. A different Danish cohort, as part of the Copenhagen General Population Study, found no association with VTE in those with hematocrit values in the top 5 percentile (hematocrit >48%; HR 1.26, 95% CI: 0.92–1.72) (50). The discrepancy between both studies might be the result of the additional covariates used in the multivariate model of the latter study. In addition to age, smoking and BMI, the model adjusted for alcohol consumption, the Charlson comorbidity index, and antiplatelet therapy. A lack of statistical power might also simply be the culprit, as reflected in the wide 95% CI. Moreover, the study found no association with arterial thrombosis in the brain (HR 1.27, 95% CI: 0.91–1.75), but did find a significantly increased risk of arterial thrombosis in the heart (HR 1.46, 95% CI: 1.06–2.00) in those with hematocrit values in the top 5 percentile. A recent retrospective cohort study examined the risk of developing major adverse cardiovascular events (MACE) or VTE in 2 cohorts of hypogonadal men who received TRT and subsequently either developed erythrocytosis (hematocrit ≥52%) or did not (51). The authors applied propensity-score matching to control for various risk factors of MACE/VTE. Those who developed erythrocytosis had a significantly higher risk of MACE/VTE than those who did not (OR 1.35, 95% CI: 1.13–1.61). Notably, there was no significant difference between groups when the threshold for erythrocytosis was set at 50%. This provides the rationale for using a cut-off of 52% for clinical-decision making.

Although insufficient data are available, it seems reasonable to assume that very high levels of hematocrit (exceeding 55–60%) might lead to substantially greater risk increases than just discussed. The degree with which blood viscosity increases with hematocrit levels depends on blood vessel diameter: increasing linearly in small vessels (the size of capillaries) and increasing exponentially in larger vessels (52).

While the aforementioned relative risks remain difficult to extrapolate to AAS users, and the absolute risk might be low – especially considering AAS users are often younger than 40–50 years of age – the risk might be compounded by other detrimental effects of AAS on blood lipids and cardiac structure and function. Thus AAS use should be strongly discouraged in those who develop polycythemia. Alternatively, therapeutic phlebotomy might be considered for those who, against better judgment, continue AAS use for prolonged periods of time. However, it remains to be determined whether therapeutic phlebotomy affects clinical endpoints such as MACE and VTE or solely ‘treats’ a laboratory abnormality. The beneficial effect of therapeutic phlebotomy on clinical outcomes in polycythemia vera patients (53) should not directly be assumed to hold true in this group of patients because of the differences in pathophysiology.

Finally, some AAS users ‘treat’ their high hematocrit levels with low-dose aspirin (acetylsalicylic acid; 75–100 mg daily). While low-dose aspirin use does not decrease hematocrit levels, it does function as an anticoagulant – purportedly negating an increased thrombosis risk. Conversely, it also increases bleeding risk, especially from gastric ulcer, and the net benefit will therefore largely depend on an individual’s cardiovascular disease (CVD) risk. Its use in primary prevention is discouraged by the 2021 ESC guidelines, with the exception of patients with diabetes mellitus at high or very high CVD risk, in whom it might be considered (54). The ESC guidelines further highlight that, even in apparently healthy persons <70 years of age with (very) high CVD risk, further studies are needed. The 2022 recommendation statement from the US Preventive Services Task Force (USPSTF) concludes that aspirin use for the primary prevention of CVD events in 40–59-year-olds with a ≥10% 10-year CVD risk has a small net benefit (55). Research in individuals with polycythemia, however, is lacking. The balance between benefit and harm can be rendered more favorable by combining aspirin use with a proton-pump inhibitor (PPI), as PPIs reduce upper gastrointestinal tract bleeding risk (56, 57). However, while PPIs are generally well-tolerated, long-term use might be associated with – sometimes serious – side effects such as increased susceptibility to gastrointestinal infections, hypergastrinaemia, micronutrient deficiencies as a result of malabsorption, and idiosyncratic reactions (58). Accordingly, these factors need to be taken into account when considering low-dose aspirin in an AAS user who appears to be at high CVD risk.

### 3.2 Acne vulgaris

Acne is a common chronic inflammatory skin condition. While its pathogenesis remains incompletely understood, it is thought that there are four pathogenic factors that are intimately involved, namely: (androgen-mediated) sebum production by the sebaceous gland, follicular colonization by *Cutibacterium acnes* (*C. acnes*), altered follicular keratinisation, and inflammation (59). Androgens play a pivotal role in sebum production as it has an absolute androgen dependency. This is supported by the observations that androgen-insensitive patients have no detectable sebum production (60), and that sebum production decreases in response to estrogen and antiandrogen administration (61). Conversely, administration of testosterone to both adult female and male subjects increases sebum production (61, 62). However, a randomized-controlled trial assigning men to receive 50, 125, 300 or 600 mg weekly of testosterone enanthate for 20 weeks found no difference in sebum production between groups (22). Given the small group sizes (12–15), lack of statistical power might have obscured any effect. One author reported dramatic hypertrophy of the sebaceous glands in skin biopsies taken from AAS users (63). Additionally, androgens are thought to play a causal role in altered follicular keratinisation, although direct evidence is lacking (64). The role of androgens in follicular colinization by *C. acnes* and in inflammation is unclear.

Acne is a commonly reported side effect by AAS users (65, 66). There does appear to be a disconnect between self-reporting of this side effect and visual examination by a physician. In the HAARLEM study, the prevalence of self-reported acne increased from 10% at the start of a cycle to 52% at the end, whereas visual examination by a physician showed a smaller increase from 13% to 29% (39). The discrepancy can be largely ascribed to AAS users classifying a few pimples as acne. The higher percentage of self-reported acne might also reflect an occurrence of this side effect at other points in time during AAS use, which would have been missed by visual examination at the end of a cycle.

To manage this side effect, some AAS users resort to ancillary drugs for treatment. This includes use of the oral prescription drug isotretinoin by a small percentage of users (65, 67). Isotretinoin is considered to be the most effective medication against acne (68). Despite its effectiveness, isotretinoin treatment is generally reserved for severe nodulocystic scarring acne or acne resistant to other therapies (68). The usual treatment in clinical practice, such as benzoylperoxide or topical retinoids, is much less often used by AAS users, possibly because they favor an oral agent that is usually very effective and easy to acquire on the black market. Isotretinoin, however, can lead to dermatologic, ophthalmologic and psychiatric/psychosomatic adverse events (69), commonly including dry skin, chapped lips, and nose bleeds (70). It is also a potent teratogen in women and therefore carries a high risk of birth defects when used during pregnancy or in the few weeks before conception. In clinical practice, dosages of 0.5–1.0 mg/kg bodyweight daily are usually prescribed. Lower dosages are commonly used by AAS users as part of their cycles. These can also be effective and demonstrate a lower frequency and severity of treatment-related side effects (71). If first-line treatment with benzoyl peroxide or a topical retinoid yields unsatisfactory results, a low dosage isotretinoin regimen under the supervision of a dermatologist can be considered in those who refuse to quit their AAS use. Treatment might also prevent acne scarring which will, obviously, be permanent even after stopping AAS use.

### 3.3 Male-pattern hair loss

Male-pattern hair loss, or androgenetic alopecia, is an androgenic condition par excellence. In the 1940s, James Hamilton described how male-pattern baldness did not develop in castrated men unless they were administered testosterone (72). Similarly, it was later described that males born with 5*α*-reductase (the enzyme responsible for conversion of testosterone into DHT) deficiency never developed male-pattern hair loss either (73). Pharmaceutical treatment of male-pattern hair loss exploits this observation through inhibition of 5*α*-reductase type 2 with finasteride (74).

The key question that remains to be answered is whether high dosages of AAS further promote the development of male-pattern hair loss. In the HAARLEM study, self-reported alopecia increased from 2% at baseline to 12% at the end of the cycle (39). The study did not include an objective measure of alopecia, which makes it difficult to distinguish between a true rise in incidence and a mere self-perceived one. Additionally, many participants used other drugs concurrently with AAS, including compounds with the potential to promote hair loss, such as thyroid hormone.

The lack of evidence notwithstanding, some AAS users resort to ancillary drugs – such as minoxidil and the 5*α*-reductase inhibitors finasteride and dutasteride – to counteract potential hair loss. While the effectiveness of 5*α*-reductase inhibitors is clear in clinical practice (75), their use in the context of high dosages of testosterone and/or other AAS is unproven and dubious at best. Any DHT-lowering effect might be easily compensated for by the increased androgenic action of supraphysiological circulating testosterone levels. Moreover, other commonly used AAS are either already 5*α*-reduced, such as methenolone, drostanolone, stanozolol, oxandrolone and oxymetholone, or do not undergo significant 5*α*-reduction in the human body, such as boldenone, trenbolone, methandienone, turinabol and fluoxymesterone (24, 76). One notable exception is nandrolone, which is converted into dihydronandrolone (DHN) by 5*α*-reductase. However, whereas testosterone is converted into the more potent androgen DHT by 5*α*-reductase (21), the conversion of nandrolone into DHN yields an androgen with significantly lower binding affinity for the AR (77, 78). Thus, whereas testosterone’s actions might be amplified in tissues expressing 5*α*-reductase, nandrolone’s actions might be diminished (21). On the basis of this metabolism, the combination of a 5*α*-reductase inhibitor with nandrolone seems particularly misguided.

### 3.4 Prostate growth and cancer

Ever since the initial isolation of testosterone by Ernst Laqueur’s group in the Netherlands in 1935 (79), and its synthesis by the Germans Adolf Butenandt and Günter Hanisch that same year (80), the prostate gland has received special attention in androgen research. In particular, an association between testosterone therapy and prostate cancer was quickly drawn based on animal experiments and limited case studies (81). Notably, the ventral prostate of the rat became the model organ for androgenic activity in the renowned Hershberger androgen bioassay, which was developed in 1953 (82). While a severely flawed approach, the bioassay remains in use today, to some extent, in the quest for selective androgen receptor modulators (SARMs) (83, 84).

Despite the initial suggestion of testosterone spurring the development of prostate cancer and the subsequent long-standing belief herein, current evidence has been unable to establish a relationship between serum androgen concentrations and prostate cancer development (85). Moreover, testosterone therapy in hypogonadal men has not been associated with an increased risk of prostate cancer (86). However, none of the trials to date have been designed to be sensitive enough to measure such an increase. Short study duration and the lack of sufficient statistical power make it impossible to draw firm conclusions. Consequently, prostate cancer in the context of high dosages of AAS remains an even larger question mark. Clinical data in the literature remain limited to a single case report describing a 40-year old chronic AAS-using bodybuilder presenting with a prostate adenocarcinoma (87). As such, there is currently no clear evidence supporting a causal relationship or association between AAS use in high dosages and prostate cancer (88). Nevertheless, large case-control studies are required to provide reasonable confidence for this.

Supraphysiological dosages of testosterone, at least up to 600 mg testosterone enanthate, did not affect serum prostate-specific antigen (PSA) levels in both healthy young (15, 22) and older men (37). Prostate volume, as assessed by magnetic resonance imaging (MRI), remained unchanged in response to graded dosages up to 600 mg testosterone enanthate weekly for 20 weeks in healthy men (22). The HAARLEM study did find a small but significant increase in PSA levels at the end of an AAS cycle compared with baseline (from 0.71 *μ*g/L to 0.93 *μ*g/L) (39). Two percent of the subjects exceeded the upper limit of the reference range (2.0 *μ*g/L). Levels dropped back to baseline 3 months after cessation of AAS use. These results are seemingly at odds with the literature that shows unchanged PSA levels in response to supraphysiological dosages of testosterone enanthate. However, previous research might not have been adequately powered to detect small differences, the various AAS used in the HAARLEM study might affect PSA levels differently, or some of the subjects might have had transiently lower PSA levels at baseline from hypogonadism caused by previous AAS use. Additionally, some research suggests that exercise might lead to a minor increase in PSA levels (89), although resistance exercise in particular has not been researched.

### 3.5 Hypertension

Hypertension is an important risk factor for the development of cardiovascular disease and end organ damage, thereby causing significant morbidity and mortality (90–92). AAS use increases blood pressure in some, but not all studies (93). The discrepancies might lie in the, commonly, small sample sizes and cross-sectional nature of these trials, which render them statistically underpowered and a less reliable source to draw cause and effect from. Perhaps the strongest data supporting an AAS-induced increase in blood pressure comes from the HAARLEM study, which enrolled 100 AAS users (46). Systolic and diastolic blood pressure increased by 7 and 3 mmHg, respectively, during AAS use. Three months after cessation of usage, blood pressure values had returned to baseline. Notably, 41% of subjects were hypertensive (>140/90 mmHg) during their cycle. Such a high prevalence of hypertension during AAS use highlights the importance of this side effect. However, because of the high prevalence of polypharmacy among AAS users, such as the use of thyroid hormone, human growth hormone and *β*-agonists, these results should be interpreted with caution.

The mechanism mediating an AAS-induced increase in blood pressure is hard to assess, and most evidence comes from *in vitro* and animal experiments. Rising blood pressure is thought to result from vasoconstriction *via* upregulation of thromboxane A_2_ expression, norepinephrine synthesis, endothelin-1 action, and activation of the renin-angiotensin-aldosterone system (RAAS) by increased angiotensin II expression (94).

The detrimental effects of these seemingly small increases in blood pressure should not be underestimated. While it is hard to estimate their impact on CVD risk, one could attempt to quantify it by looking at the – well-researched – effects of blood pressure-lowering medication. Every 10 mmHg reduction in systolic blood pressure reduces the risk of major cardiovascular events, coronary heart disease, stroke, heart failure, and all-cause mortality by 20%, 17%, 27%, 28%, and 13%, respectively (95). A persistent pharmacological increase in blood pressure – such as caused by AAS use – can be speculated to have the inverse effect.

It therefore seems prudent to monitor blood pressure in AAS users. In doing so, it is important to pay attention to blood pressure cuff size. AAS users are more likely to have large upper arm circumferences, and an inappropriately small cuff will overestimate blood pressure. The importance of using an appropriate cuff size in a muscular population was underscored in a trial examining blood pressure in a cohort of competitive bodybuilders (96). In those with an upper arm circumference greater than 33 cm, systolic blood pressure was 8.2 mmHg higher using cuff size M compared with cuff size L. As a result, 33% of the subjects would be classified as hypertensive using the – inappropriate – cuff size M, whereas only 12% would be classified as such using cuff size L. It should be noted that cuff size was adjusted according to upper arm circumference in the HAARLEM study, and thus the results were not affected by this issue (46).

While it remains to be determined if and to what extent an AAS-induced increment in blood pressure increases CVD risk, it seems prudent to discourage use when an AAS user meets the criteria for hypertension. If a patient continues using AAS long-term nonetheless, treatment seems sensible. Long-standing untreated hypertension might exacerbate the detrimental effects of AAS on cardiac structure and function, perhaps making blood pressure treatment in this population particularly relevant. The HAARLEM study, however, found no interaction between blood pressure and echocardiographic parameters – probably because the increase was mild and of relatively short duration (97). Future research is necessary to explore the efficacy of blood pressure-lowering medication in this group of patients as no trial to date has evaluated this. When considering pharmacological treatment, angiotensin-converting enzyme (ACE) inhibitors and angiotensin II receptor blockers (ARBs) might be preferrable over other blood pressure-lowering medication as they do not affect exercise capacity (98). Additionally, they are not on the current list of prohibited substances and methods of the World Anti-Doping Association, which might – ironically – be relevant for some AAS users. Alternatively, or additionally, calcium channel blockers are a preferred choice in athletes (98).

### 3.6 Hepatotoxicity

AAS are recognized to exert a detrimental effect on the liver. This particularly seems to be the case for 17*α*-alkylated AAS (11). Biochemically, this expresses itself in relatively small elevations of blood aspartate aminotransferase (AST), alanine aminotransferase (ALT), lactate dehydrogenase (LDH) and gamma-glutamyl transpeptidase (GGT) (99) values. Rarely, AAS-induced hepatotoxicity might manifest itself in jaundice and pruritus (100, 101). With such clinical presentations, elevated bilirubin values are also to be expected. Other reports in literature have also documented peliosis hepatis (102, 103), hepatocellular carcinoma (104) and adenoma (105, 106) in association with AAS use. The incidence is probably (very) low and a firm causal link has not been established.

In controlled trials, clinical signs of liver damage as a result of AAS use are indeed rare. In a double-blind randomized-controlled trial, only one of 61 HIV patients receiving a high dosage of 100–150 mg oxymetholone (a 17*α*-alkylated anabolic steroid) daily developed jaundice over the course of 16 weeks (107). In two double-blind randomized-controlled trials, hemodialysis patients received 100 mg oxymetholone daily for 24 weeks and none of these patients developed signs of liver damage (108, 109). The HAARLEM study also found no (sub)acute clinical signs of liver damage despite 67% of subjects reporting the use of oral AAS (39).

The mechanism of toxicity remains poorly understood, but it has been suggested to result from AR activation in liver cells, leading to an increased production of reactive oxygen species (ROS) (11). Therefore, AAS with sufficient resistance to hepatic breakdown and potency to activate the AR are susceptible to incur liver damage. The clinical relevance of increased biochemical markers of liver damage in response to AAS use remains unknown. Additionally, caution should be taken when interpreting these markers in AAS users. LDH, AST and ALT are expressed in skeletal muscle tissue, and their serum concentrations can remain increased for at least 7 days after intense muscular exercise such as weightlifting (110). Considering that the average training regimen of an AAS user entails at least 3 to 6 sessions per week of at least one hour, this is bound to have an effect on these markers. Therefore, ideally, measurement should be performed after at least 1 week of abstinence of exercise. GGT and bilirubin levels in serum do not appear to increase in response to exercise (111). The membrane-bound enzyme GGT is expressed in the kidneys, pancreas, spleen, lungs, brain, intestines, heart, prostate and liver – where it is mainly expressed in areas that are rich in biliary epithelial cells (112). An increase in GGT is a sensitive measure of cholestatic liver disease (113). The collective increase in these serum markers should thus be interpreted as a sign of liver damage, even in the presence of concomitant muscular exercise.

### 3.7 Dyslipidemia

Since the seminal research conducted in the 1950s as part of the Framingham Heart Study (114), the important role of lipoproteins in CVD development has become abundantly clear. Dyslipidemia, an imbalance in these lipoproteins, is recognized as an important risk factor for CVD, and treatment thereof forms one of the cornerstones of primary and secondary CVD prevention. Plasma low-density lipoprotein (LDL)-cholesterol levels are positively associated with CVD risk and causality has been firmly established (115). High-density lipoprotein (HDL)-cholesterol levels are inversely associated with CVD risk, although a causal role remains doubtful (116).

While not seen in every clinical trial, treatment of hypogonadal men with testosterone therapy reduces circulating HDL-cholesterol (117). Similar changes are seen in men receiving supraphysiological dosages (200–600 mg weekly) of testosterone enanthate (15, 37, 118, 119), although not all trials show a statistically significant decrease (34, 120, 121). Notably, in trials administering nandrolone decanoate (100–200 mg weekly) HDL-cholesterol remains unchanged (122–124). While this might indicate a true difference compared with testosterone, it might also be attributed to the relatively low dosages used and small samples sizes that make the research liable to type II statistical errors (a ‘false negative’). Interestingly, decreases in HDL-cholesterol are an ubiquitous finding with the administration of 17*α*-methylated oral AAS – even in low dosages (118, 121, 125–128). It is appealing to assume that the increased exposure of the liver to this class of AAS is the culprit. In the HAARLEM study, HDL-cholesterol decreased by 0.4 mmol/L during use and returned to baseline 3 months after cessation of use. Notably, two-thirds of the subjects used oral AAS during their cycle, the use of which was associated with a more adverse lipid profile (46).

It is thought that AAS impact HDL-cholesterol by increasing hepatic lipase (HL) activity (118–121). The enzyme acts as a lipase on phospholipids and triglycerides in lipoprotein particles, including HDL. It converts the larger HDL_2_ particles into smaller HDL_3_ particles (129). However, administration of a low dosage (6 mg daily) of stanozolol (a 17*α*-alkylated anabolic steroid) for 2 weeks reduced HDL-cholesterol levels by 20% in 2 HL-deficient brothers (130). The decrease was less than that of 2 control subjects in the same study who experienced a reduction of 49%. The small sample size calls for caution when interpreting these results which suggest that increased HL activity is only partly responsible for the AAS-induced decrease in HDL-cholesterol. Other factors involved remain unclear. Regardless of the mechanism of action, it is uncertain how an AAS-induced decrease in HDL-cholesterol might affect CVD risk. As noted earlier, while its levels are inversely associated with CVD risk, a causal role remains doubtful. Indeed, ‘drugging’ HDL-cholesterol upwards with cholesteryl ester transfer protein (CETP-)inhibitors has, unfortunately, failed to reduce CVD (131–134). Conversely, ‘drugging’ it downwards should not automatically be assumed to increase CVD risk, although it may well be the case (116).

The lack of an apparent causal relation between HDL-cholesterol levels and CVD risk has driven research into HDL function. In particular, cholesterol efflux capacity (CEC): the capacity of HDL particles to function as a cholesterol acceptor and thereby facilitate removal of cholesterol from cells such as lipid-laden macrophages (foam cells) within the intima of arteries (135). Similar to HDL-cholesterol levels, higher CEC is associated with lower atherosclerotic CVD risk (136). Whether a pharmacological increase in CEC also reduces CVD risk remains to be determined.

Limited research has investigated the effect of AAS on CEC. A trial in older hypogonadal men randomized to TRT with or without the 5*α*-reductase inhibitor dutasteride noted no change in CEC (137). CEC also remained unchanged in transgender males (female to male) undergoing treatment with testosterone gel or injections (138). In contrast, cross-sectional research demonstrated impaired CEC in AAS users compared with age-matched, strength-trained nonusers and sedentary controls (139). It is possible that the higher dosages used, use of various AAS, or different methods used to measure CEC (which is not standardized), might underlie these differences.

LDL-cholesterol is generally unaffected by injectable AAS such as testosterone enanthate (up to 600 mg weekly) (15, 34, 37, 119–121) and nandrolone decanoate (up to 200 mg weekly) (123, 124), with one trial showing a 16% decrease after 6 weeks of 200 mg testosterone enanthate weekly (118). Oral AAS again demonstrate unfavorable changes, consistently increasing LDL-cholesterol (38, 118, 121, 125, 126). In the HAARLEM study, LDL-cholesterol increased by 0.45 mmol/L compared with baseline (46).

In contrast with the adverse effect on LDL- and HDL-cholesterol, AAS appear to exert a neutral or favorable effect on lipoprotein (a) (Lp(a)). Elevated Lp(a) levels are considered an established causal risk factor for CVD (140). In a double-blind trial, nandrolone decanoate (200 mg weekly) for 8 weeks decreased Lp(a) compared with baseline, but not compared with placebo, in a group of bodybuilders (124). In the same publication, a second nonblinded trial is described in which AAS users self-administer their own cycle. Herein, Lp(a) plummeted (-83%) compared with baseline after 8 weeks, with partial return to baseline 6 weeks after cessation of AAS use. However, 600 mg testosterone enanthate weekly for 3 weeks did not change Lp(a) in elderly obese men (119), yet 200 mg testosterone enanthate weekly for 3 weeks decreased Lp(a) substantially by 38% compared with baseline in healthy male weightlifters – although a placebo group was lacking (141). Finally, in the HAARLEM study Lp(a) decreased by almost 50% at the end of an AAS cycle and returned to baseline 3 months after cessation of use (46). Regardless, the benefit of therapeutically decreased Lp(a) on CVD risk remains unclear (142) and might only be potentially beneficial for those with elevated Lp(a) levels that correlate with increased CVD risk, which encompasses 15–20% of the population (143). Therefore, it should not be assumed that an AAS-induced decrease in Lp(a) might negate the other effects that are detrimental to cardiovascular health.

As with other side effects, some AAS users self-medicate to mitigate this unfavorable shift in lipid profile. This includes both dietary supplements, such as niacin, red yeast rice extract and berberine, but also prescription medication such as statins. Niacin raises HDL-cholesterol, but has no effect on overall mortality, cardiovascular mortality, non-cardiovascular mortality, the number of fatal or non-fatal myocardial infarctions, nor the number of fatal or non-fatal strokes (134). Besides its side effects, its use might lead to underestimation of CVD risk when using risk algorithms that are guided by HDL-cholesterol levels. As such, its use should be discouraged. Red yeast rice extract contains monacolin K (lovastatin), which explains its efficacy in reducing LDL-cholesterol (144). While its use is likely safe (145), commercial red yeast rice supplements can vary considerably in their lovastatin content (146). Berberine also reduces LDL-cholesterol, although this finding is mostly supported by trials of poor methodological quality (147). Drug-drug interactions might be looming with berberine supplementation, as it can inhibit the activity of several enzymes belonging to the cytochrome P450 superfamily (CYP2D6, CYP2C9 and CYP3A4) and affects dextromethorphan, midazolam and losartan pharmacokinetics (148).

With the exception of its effect on Lp(a), AAS use – especially use of 17*α*-alkylated AAS – leads to a more atherogenic lipid profile (see **Table 1**). This might help explain the results of a population-based cohort study in which men that tested positive for AAS had twice the cardiovascular morbidity and mortality rate as those who tested negative (149). Indeed, in a cross-sectional study comparing AAS users with nonusers, a higher coronary artery plaque volume was found in the former, and all angiographic measures of coronary pathology showed a strong association with lifetime duration of use (150). Nevertheless, it should be acknowledged that causality cannot be drawn from such studies and further research is necessary to better explore this detrimental avenue of AAS use on health.

**Table 1:** Effects of injectable and 17*α*-alkylated AAS on serum lipoproteins and cholesterol efflux capacity.

AAS type	LDL-C	HDL-C	CEC	Lp(a)
Injectable AAS	↔	↓	↓	↓↓
17α-alkylated AAS	↑↑	↓↓		

We do not distinguish between injectable and 17*α*-alkylated AAS for CEC and Lp(a) due to lack of data differentiating between the two.↔, no change; ↓, small to moderate decrease; ↓↓, moderate to large decrease; ↑, small to moderate increase; ↑↑, moderate to large increase. Abbreviations: AAS, anabolic–androgenic steroids; LDL-C, low-density lipoprotein cholesterol; HDL-C, high-density lipoprotein cholesterol; CEC, cholesterol efflux capacity; Lp(a), lipoprotein (a).

It seems appropriate to manage dyslipidemia in (long-term) AAS users according to current guidelines (151) just as in any other patient. In doing so, a few things need to be considered. Risk estimation using algorithms such as SCORE2 might underestimate risk because certain side effects of AAS use, such as the detrimental changes to cardiac structure and function they elicit, could act as risk modifiers. Echocardiographic proof of such changes might therefore aid in ‘grey zone’ risk estimation situations. The use of HDL-cholesterol boosting supplements, such as niacin, could also lead to underestimating risk when using algorithms based on HDL-cholesterol. Thus it is important to inquire about supplement use in this group of patients. If pharmacological intervention is indicated, statins are the first-line of treatment to lower LDL-cholesterol. Statins might cause muscle pain in a small percentage of users (152), but this side effect might occur more frequently in those who engage in regular intense exercise (153). For this group of patients, an expert panel recommends the use of hydrophilic statins (rosuvastatin and pravastatin) at a low-to-moderate dose, as hydrophilic statins are thought to be more hepatoselective (153). If the statin is not tolerated, it is advisable to change to a lipophilic statin (e.g. simvastatin or atorvastatin), reduce the dose, or try an alternate-day regimen (154). If despite these attempts statin intolerance remains an issue, other pharmacological options, such as ezetimibe or proprotein convertase subtilisin/kexin type 9 (PCSK9) inhibitors, should be explored.

### 3.8 Nephrotoxicity

There is no good-quality evidence indicating that AAS use is damaging to the kidneys. However, some findings in the literature point to a potential detrimental effect. In the HAARLEM study, a transient small increase in serum creatinine concentrations of unknown clinical relevance was observed during AAS use (from 93.1 *μ*mol/L (1.05 mg/dL) to 97.8 *μ*mol/L (1.11 mg/dL)). Creatinine concentrations returned to baseline after cessation of use. Albuminuria, as measured by dipstick analysis, emerged or increased in 16% of the subjects (155). A larger increase in serum creatinine levels was observed in a small 4-week placebo-controlled trial with resistance-trained men randomized to 330 mg daily of the oral prohormone 3*β*-hydroxy-5*α*-androst-1-en-17-one (1-androsterone) or placebo (38). Bioactivation of the prohormone into the potent anabolic steroid 17*β*-hydroxy-5*α*-androst-1-en-3-one (1-testosterone) results from oxidation at carbon 3 of the A-ring and reduction at carbon 17 of the D-ring of the steroid nucleus (156). In those receiving 1-androsterone, serum creatinine levels increased significantly from 97.3 *μ*mol/L (1.1 mg/dL) to 115.0 *μ*mol/L (1.3 mg/dL).

Serum creatinine levels are commonly used to estimate the glomerular filtration rate (eGFR) using formulas such as the Chronic Kidney Disease Epidemiology Collaboration (CKD-EPI) equation (157). The eGFR is a good and independent predictor of all-cause and cardiovascular mortality and kidney failure in a wide range of populations (158). Nevertheless, it should be appreciated that the accuracy of the equation is predicated on the assumption that serum creatinine levels accurately reflect the GFR – which is doubtful in this particular population. Creatinine is, for the most part, a product of the spontaneous nonenzymatic degradation of creatine (Cr) and creatine phosphate (PCr) (159). The overall conversion rate for the total Cr pool (Cr + PCr) is (almost) constant and approximately 1.7% daily. Given that nearly all of the body’s creatine is stored in skeletal muscle, an increase in muscle mass increases the daily production of creatinine and can subsequently elevate serum creatinine levels without impacting GFR. The eGFR based on serum creatinine levels is therefore an underestimate in muscular populations.

Additionally, there is some evidence indicating that AAS use might increase endogenous creatine production. Administration of the oral anabolic steroid 17*α*-methyltestosterone increases urine excretion of creatinine and guanidinoacetic acid (160). This suggests that it increases arginine:glycine amidinotransferase (AGAT) expression: the enzyme responsible for the rate-limiting step in creatine biosynthesis by forming guanidinoacetic acid – the direct precursor of creatine (161). While direct data are lacking, these data suggest that 17*α*-methyltestosterone increases creatine biosynthesis and consequently the total creatine pool. Creatine is also used as a dietary supplement to increase muscle creatine stores (162). Notably, the dietary supplement creatine ethyl ester can lead to markedly increased serum creatinine levels (163, 164), probably as a result of rapid degradation into creatinine in aquatic media with near-neutral pH (165). Collectively, these factors make the interpretation of – especially small – changes in creatinine-based eGFR challenging.

As suggested by Baxmann et al. (166), measuring serum cystatin C might be more reliable to estimate GFR in healthy individuals with higher muscle mass and potential mild kidney impairment. Cystatin C is a protein that is produced by all nucleated cells and is freely filtered at the glomerulus. Compared with serum creatinine, serum cystatin C concentrations are less affected by age, sex, race, and, most importantly, muscle mass (167). One study to date has investigated the effect of high dosages of AAS on serum cystatin C concentrations (168). Serum creatinine and cystatin C concentrations were measured in 57 current AAS users, 28 past users, and 52 non-AAS-using weightlifters. Both parameters were significantly higher in current users than in nonusers. Unfortunately, because of its cross-sectional setup, this study does not allow to infer causality. If the increase indeed is causal, it remains to be determined whether this reflects a true decrease in GFR or whether AAS affect serum cystatin C concentrations by other means. Future research might help answer this question by comparison with more accurate (though less convenient) GFR filtration markers such as iohexol or iothalamate.

A few reports in the literature have linked AAS use to focal segmental glomerulosclerosis (FSGS) (169–171). FSGS is a histopathological finding marked by glomerular lesions, mediated by diverse insults directed to, or inherent within, the podocytes (172). Herlitz et al. were the first to present a case series of nine patients who developed FSGS after prolonged AAS use (169). A tenth patient described in their publication had no discrete lesions of segmental sclerosis but did have glomerulomegaly. All patients presented with proteinuria (mean 10.1 g/d; range 1.3–26.3 g/d) and all but one presented with elevated serum creatinine levels (mean 265.3 *μ*mol/L (3.0 mg/dL); range 115.0–689.7 *μ*mol/L (1.3–7.8 mg/dL)). Mean body mass index (BMI) was 34.7 kg/m^2^, with all but one of the patients having a BMI of ≥30 kg/m^2^. Follow-up data were available for eight of the patients, of which one progressed to end-stage renal disease within 1 month of biopsy. The remaining seven patients either stabilized or showed a decrease in serum creatinine levels and proteinuria after starting medical treatment (in the form of ACE inhibitors, ARBs, and/or renin inhibitors) and stopping AAS use. One of the patients resumed AAS use and subsequently developed progressive renal insufficiency and an increase in proteinuria. It is appealing to speculate that a very high (lean) body mass, perhaps in combination with very high dietary protein intake (as is common in this population), shapes a permissive environment for the development of FSGS by chronic AAS use.

Whether or not AAS are eventually found to impair kidney function, long-term use leading to (untreated) hypertension is most likely to inflict renal damage.

### 3.9 Testosterone deficiency

**Figure 5:**
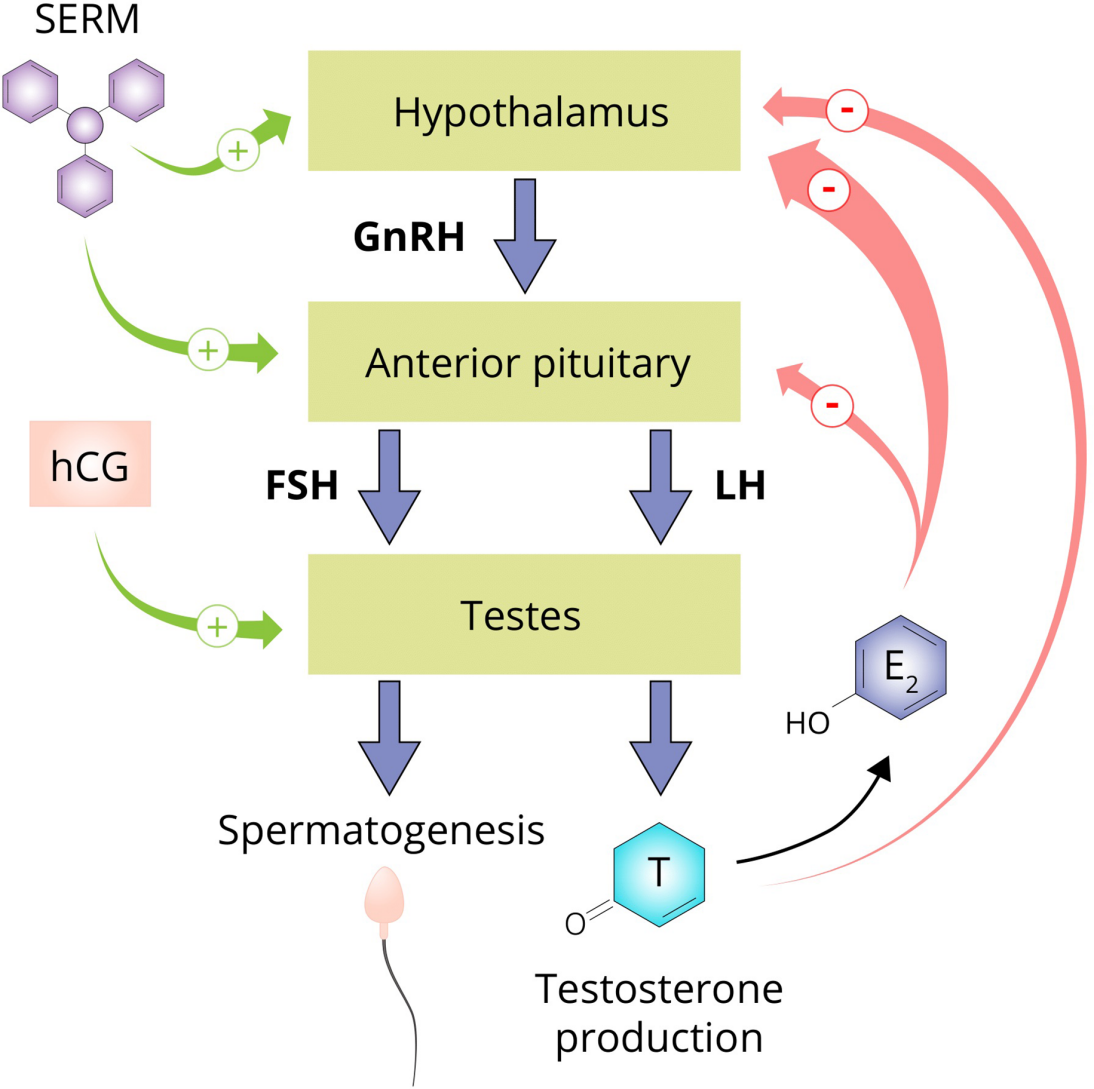
The hypothalamus secretes GnRH which stimulates the anterior pituitary to secrete the gonadotropins LH and FSH. LH stimulates the Leydig cells of the testis to produce testosterone and in conjunction with FSH, regulates spermatogenesis. Testosterone suppresses its own production directly by exerting negative feedback on the hypothalamus and by conversion to estradiol on both the hypothalamus and anterior pituitary. SERMs are capable of negating the negative feedback imposed by estrogens and are therefore commonly used by AAS users to supposedly aid in recovery of testosterone production after an AAS cycle (‘post-cycle therapy’). hCG is able to directly stimulate the testis to produce testosterone by binding and activating the luteinizing hormone/choriogonadotropin receptor (LHCGR) which it shares with LH. Abbreviations: SERM, selective estrogen receptor modulator; hCG, human chorionic gonadotropin; GnRH, gonadotropin-releasing hormone; FSH, follicle-stimulating hormone; LH, luteinizing hormone; T, testosterone; E_2_, estradiol.

The testicular production of testosterone is governed by the hypothalamic–pituitary–gonadal axis (HPGA; see **Figure 5**). Gonadotropin-releasing hormone (GnRH) neurons of the hypothalamus secrete GnRH in pulsatile fashion into capillaries of the hypophyseal portal system. GnRH binds to its receptor, the GnRH receptor, on gonadotrophic cells of the anterior pituitary. Activating this G protein-coupled receptor triggers a cascade of events that stimulates the synthesis and release of luteinizing hormone (LH) and follicle-stimulating hormone (FSH). LH and FSH, in turn, bind to their cognate receptors on the Leydig cells and Sertoli cells of the testis, respectively. LH stimulates testosterone production and, in conjunction with FSH, regulates spermatogenesis. Testosterone, and its estrogenic metabolite estradiol, exert negative feedback on the hypothalamus and pituitary to suppress their own synthesis (173, 174). Estradiol in particular is extraordinarily potent at suppressing gonadotropin secretion as, on a molar basis, it is estimated to be 200-fold more potent than testosterone in doing so (175). Consequently, exogenously administered AAS will also exert negative feedback, thereby suppressing testicular testosterone production and spermatogenesis. This suppression persists for some time after cessation of use. The time course and factors affecting HPGA recovery after cessation of use are poorly characterized. Lingering low testosterone levels might be explained by prolonged release of esterified AAS or their metabolites, prolonged suppression of SHBG resulting in increased testosterone clearance, continued undisclosed AAS use, or transient failure of the hypothalamus or pituitary to adequately resume hormone production for unknown reasons (‘AAS-induced hypogonadism’).

Some initial data regarding the time course of HPGA recovery after AAS use have recently been published (176). When gonadal function was normal before an AAS cycle, there was a 90% chance of having normal testosterone levels 3 months after cessation and a 100% chance at the end of follow-up (1 year after the start of the cycle). At the group level, mean testosterone levels returned to baseline 3 months after cessation. Importantly, 37% of the subjects had signs of abnormal gonadal function at baseline of which 95% had a history of AAS use. This might be explained by incomplete recovery of the HPGA due to recent AAS use or prolonged or persistent hypogonadism from past use. A case-control study also suggests that AAS use leads to a persistent small reduction in testosterone levels (177). However, recent or current unreported AAS use, reverse causality and other factors inherent to a case-control study design make it difficult to ascertain a true cause-and-effect relationship. Regardless, persistent AAS-induced hypogonadism has been reported in the literature in several cases (65, 178). Future research is needed to delineate the AAS cycle features or patient characteristics that hinder recovery and result in partially reversed, prolonged, or persistent hypogonadism.

AAS users commonly use ancillary drugs to aid HPGA recovery. This unproven practice is known as post-cycle therapy (PCT) and usually includes selective estrogen receptor modulators (SERMs) such as tamoxifen and clomiphene, aromatase inhibitors such as exemastane, letrozole and anastrozole, and human chorionic gonadotropin (hCG). SERMs negate the negative feedback on the pituitary exerted by estrogens, and aromatase inhibitors impede the formation of these estrogens. Both classes of compounds indeed increase testosterone levels in men with hypogonadism due to various causes. Testosterone levels decrease again after the agents are discontinued, implying that they do not solve the underlying cause of hypogonadism. As such, it remains to be seen whether they show efficacy in the case of AAS-induced hypogonadism, as PCT is usually performed for a few weeks. In the HAARLEM study, testosterone levels were similar 3 months after cessation of AAS use in those who did and did not perform PCT, but a small beneficial effect within this time frame could not be excluded (46). Finally, hCG directly stimulates the testes to produce testosterone by binding to the luteinizing hormone/choriogonadotropin receptor (LHCGR) which it shares with LH. This could lead to continued suppression of LH and FSH levels when employed as PCT, but is assumed by AAS users to aid in recovery of testicular function. This might be probable in select cases which demonstrate biochemical evidence of primary hypogonadism (elevated gonadotropin levels with low testosterone levels), but evidence is lacking.

### 3.10 Infertility

Just like testicular testosterone production, spermatogenesis is governed by the HPGA. The concerted action of LH and FSH on the testes stimulates spermatogenesis, and suppression of these hormones inhibits it. FSH acts directly on spermatogenesis by activating FSH receptors on Sertoli cells, whereas LH works indirectly through stimulating testosterone production by activating LHCGRs on Leydig cells, which in turn activates ARs on Sertoli cells (179). Despite testosterone being the primary mediator of LH’s effect on spermatogenesis, exogenous administration of testosterone cannot support spermatogenesis. Intratesticular testosterone (ITT) levels are about 50 to 100 times higher than in circulation (180) and exogenous administration severely suppresses this to levels that are unable to support spermatogenesis (181).

The premise of hormonal male contraception hinges on the negative feedback exerted by sex hormones on LH and FSH secretion. Interestingly, even a dosage that is roughly twice that of TRT (200 mg testosterone enanthate weekly) only partially suppresses LH (-66.7%) and FSH (-62.5%) and, indeed, leads to azoospermia in only about two out of three men (182). As such, efforts have been made to complete gonadotropin suppression by adding progestins. While the addition of a progestin leads to almost undetectable gonadotropin levels, and consequently to azoospermia or severe oligozoospermia in the vast majority of men, a small percentage of men remain potentially fertile (183, 184). One reason for this might be that the low intratesticular testosterone levels derived from the circulation continue to stimulate spermatogenesis in some men (180). In the HAARLEM study, nearly all subjects had undetectable LH and FSH levels during AAS use. However, only two-thirds of subjects were azoo- or oligozoospermic at the end of their cycle (176). There was no association between the duration of the AAS cycle and the degree of suppression of spermatogenesis. Given that 12% of subjects reported use of hCG on-cycle, and hCG can preserve some spermatogenesis during gonadotropin suppression (185), a slightly lower fraction of men might have developed azoo- or oligozoospermia in this study than expected.

Suppression of spermatogenesis leads to testicular atrophy, which is mainly a cosmetic issue, although some men subsequently complain of retractile testes. The seminiferous tubule compartment of the testis, which hosts spermatogenesis, occupies about two-thirds of its volume (186). A considerable fraction of this compartment consists of developing sperm cells. Consequently, spermatogenesis arrest results in testicular atrophy. Sex steroid-induced suppression of spermatogenesis reduces testicular volume by 16.5–30.0% (176, 182, 183). Some AAS users might, therefore, resort to the use of hCG or human menopausal gonadotropin (hMG) to maintain testis volume rather than seeking to preserve fertility *per se*.

Detailed data regarding the time course and extent of spermatogenesis recovery are available for hormonal male contraceptive use. An integrated multivariate time-to-event analysis of 30 hormonal male contraceptive studies showed a median of 5.4 months for sperm count to return to the individual baseline value, with 54% of subjects recovering within 6 months, 83% within 12 months, 95% within 16 months, and 100% within 24 months (187). While suppression of spermatogenesis by hormonal male contraception and AAS use share the common mechanism of sex steroid-induced gonadotropin suppression, some caution should be taken when extrapolating these figures to AAS users. Since AAS users commonly inject esterified AAS with long half-lives in large doses, it will take at least several weeks before recovery starts. Some research has specifically looked at the time to recovery of spermatogenesis after AAS use. A cross-sectional observational study comparing current AAS users with past users and nonusers tried to quantify the mean time to recovery of sperm output and concentration (188). The authors calculated the mean time to recovery by calculating the time taken to reach the mean value of the nonusers group according to a linear regression of the variable over time since cessation of AAS use. Mean time to recovery of sperm output and concentration were 14.1 and 10.4 months, respectively. In the HAARLEM study, mean sperm concentration decreased from 46.8 million/mL to 11.7 million/mL during AAS use. 68% of subjects met the criteria for oligozoospermia or azoospermia based on their sperm concentration (<15 million/mL). A substantial number of subjects (28%) already met this criteria at baseline. This is considerably higher than might be encountered in the general population and is suggestive of incomplete recovery of spermatogenesis from previous AAS use. Three months after cessation of use, mean sperm concentration was still significantly lower compared with baseline.

### 3.11 Erectile dysfunction

Erectile dysfunction is defined as the inability to achieve or maintain an erection sufficient for satisfactory sexual performance (189). Testosterone plays an important role in nearly every aspect of erectile function (190) and erectile dysfunction is considered a suggestive symptom of testosterone deficiency (191). However, not all hypogonadal men develop erectile dysfunction, and TRT generally leads to only a small improvement in erectile function (192). Importantly, not all men with erectile dysfunction are hypogonadal.

Erectile dysfunction can have various causes. The nature of erectile dysfunction can be classified as psychogenic, organic or mixed psychogenic and organic (193). Because of the intimate role of testosterone in erectile function, erectile dysfunction can develop as a post-cycle side effect of AAS use. However, it should be kept in mind that erectile dysfunction in an AAS user is not necessarily the result of AAS use *per se* and might be a symptom of an underlying psychiatric disorder. The presence of rigid morning or night erections, sudden onset, intermittent course and short duration are a good indication of a psychogenic cause. Lack of morning or night erections, a gradual onset, progressive course, or long duration suggest an organic cause.

AAS users somewhat commonly experience erectile dysfunction (65), with 8% of subjects in the HAARLEM study reporting it at baseline and 12% reporting to have experienced it during AAS use. Three months after ceasing AAS use and 1 year after the start of the AAS cycle it was reported by 14% and 1% of users, respectively. None of these changes were significantly different from baseline, which might be a type II error. The relatively high percentage of users reporting erectile dysfunction at baseline compared with the last follow-up measurement suggests this side effect might have still been present from relatively recent AAS use at baseline in some. However, since not all AAS users completed follow up, attrition bias might also (partly) explain the difference. Regardless, erectile dysfunction might develop after an AAS cycle as a result of the transient hypogonadal state. In this case a loss of libido due to testosterone deficiency usually underlies the erectile dysfunction. Not uncommonly in this time period, dissatisfaction with the intercourse, frustration of the sexual relationship with the bed partner(s), and loss of self-confidence may lead to perpetuation of erectile dysfunction even when testosterone levels recover. Erectile dysfunction is also sporadically reported during AAS use. The mechanism for this is unclear, but, given that estradiol, independently of testosterone, also plays a role in regulating erectile function (194), it might involve an imbalance between androgenic and estrogenic action. Erectile dysfunction may also be a consequence of psychological factors, as libido may rise sharply in an AAS user during the cycle and occasionally hinder a healthy and mutual sexual relationship.

Some AAS users self-medicate with phosphodiesterase type 5 (PDE5) inhibitors such as sildenafil to counteract erectile dysfunction (65). This class of drugs inhibits the enzyme PDE5 which breaks down cGMP – the second messenger molecule responsible for conveying the signal of the cavernous nerve to induce an erection (195). PDE5 inhibitors are the mainstay drug in erectile dysfunction treatment and are generally tolerated well, providing satisfactory results. Side effects include headache, flushing, dyspepsia, nasal congestion, dizziness, transient abnormal vision and cyanopsia (specific to sildenafil), and back pain and myalgia (specific to tadalafil) (196). While these drugs are commonly already acquired by AAS users from the black market, they might be prescribed to patients suffering from erectile dysfunction which is either organic or psychogenic in nature. A referral to a sexologist is advised for those in whom a psychogenic cause is likely – which is common in our experience.

### 3.12 Gynecomastia

Gynecomastia is the benign enlargement of the glandular tissue of the breast (197). The condition is not to be confused with pseudogynecomastia, also referred to as lipomastia or adipomastia, in which excessive adipose tissue gives the appearance of gynecomastia. Gynecomastia often develops during puberty, with a reported peak incidence of 65% in 14-year-old boys (198). The condition remains prevalent throughout adulthood, with one study reporting gynecomastia in 40.5% of healthy young men aged 18–26 years (199) and another reporting detectable palpable breast tissue in 36% of healthy adult men aged 16–58 years (200). While palpable, in the great majority of cases the gynecomastia was small in size.

The root cause of gynecomastia is hormonal, resulting from an imbalance of androgenic and estrogenic action on breast tissue (201). More specifically, gynecomastia results from an absolute or relative deficiency of androgenic, or absolute or relative excess of estrogenic, action on breast tissue. A variety of conditions that affect the levels or actions of these sex hormones can therefore cause gynecomastia. Not surprisingly, gynecomastia is a side effect that can occur as a result of AAS use. In an uncontrolled multicenter contraceptive efficacy study, 271 men received 200 mg testosterone enanthate weekly for a minimum of 6 months (202). During this intervention, only 9 men (3%) developed gynecomastia. In contrast, the prevalence of gynecomastia increased from 7% at baseline to 19% at the end of an AAS cycle in the HAARLEM study (39). Almost all of them had Simon grade 1 gynecomastia, with one subject progressing from Simon grade 2 at the end of the AAS cycle to grade 3 three months after the cycle, presumably due to the hypogonadal state that followed after cessation of use.

Since large doses of AAS are administered during an AAS cycle, it is evident that the development of gynecomastia during AAS use is not the result of an absolute or relative deficiency of androgenic action. Estradiol levels increase dose-dependently with testosterone administration; however, the increase is of proportionately lesser magnitude with increasing doses, indicating saturation of aromatase activity (23). As such, it seems reasonable to conclude that an absolute excess of estrogenic action causes the development of gynecomastia during AAS use, regardless of its relative action compared with androgens.

Among AAS users there is the belief that AAS might cause gynecomastia through alternative pathways, such as increased progestin action at the mammary glands or increased prolactin levels. While gynecomastia can develop in patients with hyperprolactinemia, the condition arises secondary to the gonadotropin suppression prolactin can cause (203). Importantly, prolactin levels are suppressed by androgens (204). Despite the lack of any evidence or even theoretical basis for this practice, some AAS users self-medicate with dopamine agonists such as bromocriptine and cabergoline to lower their assumed increased prolactin levels and treat their ‘prolactin-induced’ gynecomastia. Such practice should be discouraged because it is illogical and produces possible side effects such as cardiac abnormalities or arrhythmia. Increased progestin action at the mammary gland is also very unlikely to be a cause of AAS-induced gynecomastia. AAS do not increase progesterone levels and only a select few demonstrate significant progesterone receptor activation (205). Moreover, no gynecomastia was noted in a 6-month hormonal male contraception study combining administration of testosterone enanthate with the potent progestin levonorgestrel (0.5 mg daily) (183).

While not approved for treatment of gynecomastia, anti-estrogenic drugs such as aromatase inhibitors and SERMs are prescribed off-label and used in clinical trials to treat gynecomastia. AAS users also self-medicate with these drugs to either prevent gynecomastia from developing or to reduce the size of existing gynecomastia. For unclear reasons, the efficacy of aromatase inhibitors for treating gynecomastia of various causes appears to be poor or modest at best (206–209), although anastrozole successfully treated two cases of TRT-associated gynecomastia (210). In contrast, the SERM tamoxifen in dosages of 10–20 mg daily for several months has shown efficacy in up to 90% for the resolution of gynecomastia (201) with another review noting partial regression in 80% of patients and complete regression in about 60% (197). A few AAS users treat their gynecomastia with the SERM raloxifen, although data on it are scarce with only a single retrospective chart review published in the scientific literature (211).

### 3.13 Cardiomyopathy

A Swedish national population-based cohort study found a cardiovascular morbidity and mortality rate twice as high in individuals who tested positive for AAS use compared with those who tested negative (149). Similarly, a Danish retrospective matched cohort study found non-ischaemic heart disease rates, such as cardiomyopathy and atrial fibrillation, to be three times higher in those who tested positive for AAS use compared with matched controls (212).

The association between cardiomyopathy and AAS use has long been controversial (213) as a result of mixed study findings, mainly due to small sample sizes, weak study design and insensitive measurements of older echocardiographic techniques. For example, the first clinical trial examining the effects of AAS use on the heart was published in 1985 (214). Using a cross-sectional study design, Salke et al. performed measurements using M-mode echocardiography in two small groups of bodybuilders: one self-administering AAS and the other denying to have ever used AAS. They also examined a control group of inactive individuals. Although a few measurements (such as left posterior wall and interventricular septum thickness) were significantly different in the bodybuilders compared with controls, no significant differences were found between both groups of bodybuilders.

Technological advances have led to more sensitive measurements of cardiac structure and function. Using standard Doppler echocardiography and pulsed tissue doppler imaging (TDI), Nottin et al. found impaired left ventricular (LV) relaxation, as evidenced by a prolonged isovolumetric relaxation time and smaller peak E_m_/A_m_ ratios at the mitral annulus, in AAS-using bodybuilders compared with controls (215). Similar results were reported by Krieg et al. who observed a decreased E_m_/A_m_ ratio on the basal part of the interventricular septum in a small group of AAS-using bodybuilders compared with steroid-free strength athletes and sedentary controls (216). E_m_/A_m_ ratio is the ratio between the velocity of the myocardial wall during early diastole (E_m_) and late diastole (A_m_), and a decrease in this ratio is indicative of diastolic dysfunction.

A particularly comprehensive cross-sectional trial was published in 2017 and authored by Baggish et al. (150). A total of 140 experienced male weightlifters were subjected to echocardiography. Of these 140 men, 86 reported ≥2 years of cumulative lifetime AAS use, and 54 reported no use of AAS. The group of 86 men could be further subdivided into those using AAS at the time of the study and those who were not. Main findings included a substantial impairment of LV systolic function in the AAS group compared with the nonusers as evidenced by an 11%-point lower LV ejection fraction (LVEF) and impaired longitudinal 4-chamber strain (+4.6). Over half the subjects in the AAS group had an LVEF below the reference range (<52%). Interestingly, 71% of subjects below the reference range were using AAS at the time of the study, and there was no statistically significant difference in LVEF between those not using AAS at the time and the AAS-naive individuals. This suggests that, at least to some extent, the change in LVEF might be reversible. Early LV relaxation velocity (E´) was also impaired, with half of those using AAS at the time falling below the normal threshold of 8.5 cm/s, and those off-cycle showing partially normalized values. LV mass, also after correction for body surface area, was significantly higher in the AAS group compared with the AAS-naive subjects.

**Figure 6:**
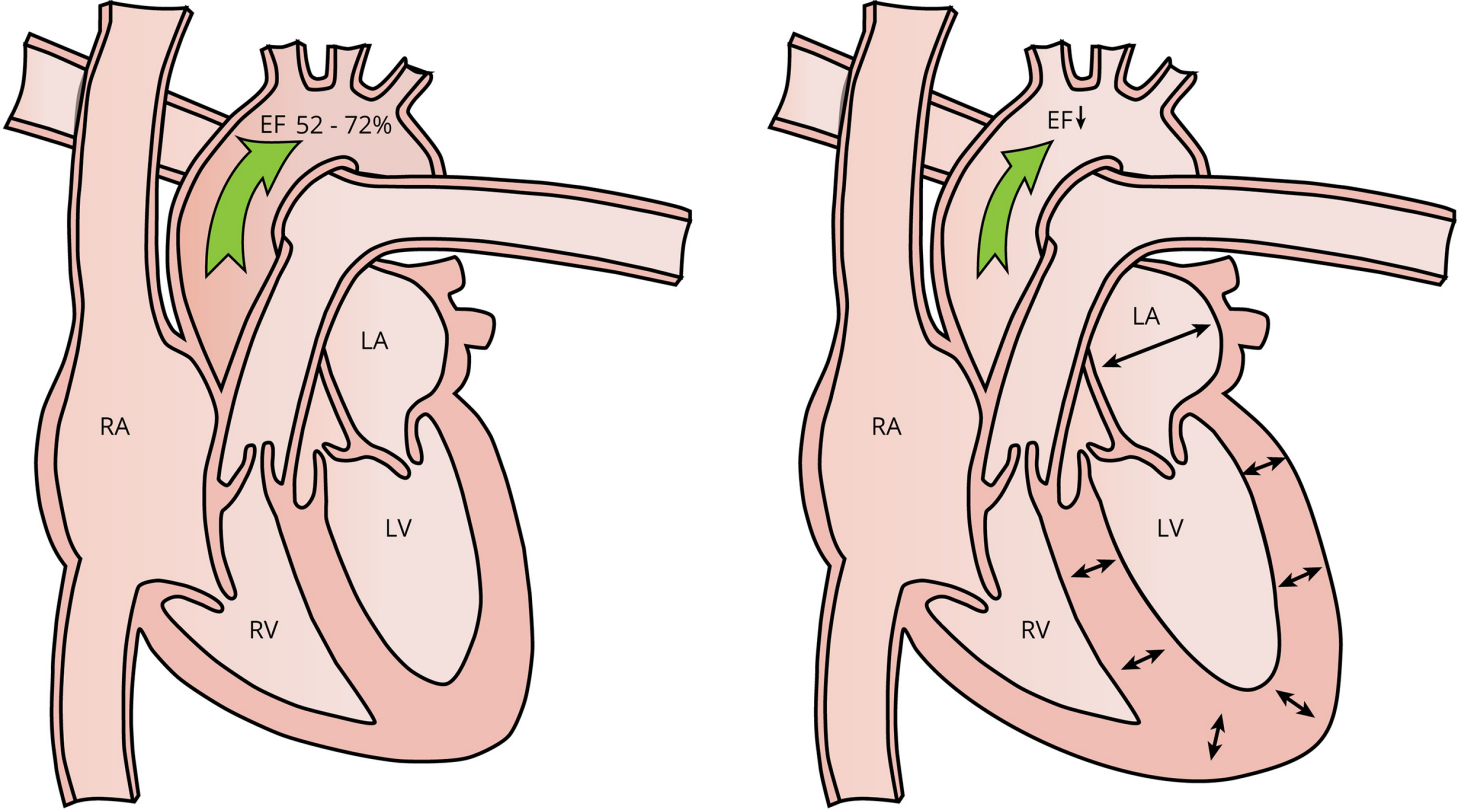
Left a normal heart and right the remodeling induced by AAS use. AAS use can lead to concentric left ventricular hypertrophy, as signified by an increased LV posterior wall and interventricular septum thickness. Additionally, ejection fraction is decreased and left atrium size might increase. Abbreviations: EF, ejection fraction; RA, right atrium; LA, left atrium, RV, right ventricle; LV, left ventricle.

Thirty-one men enrolled in the HAARLEM study were subjected to 3D echocardiography before, at the end, and a median of 8 months after the start of their self-administered AAS cycles (97). The prospective nature, comprehensive evaluation and long follow-up make the results of this trial particularly interesting. The findings were largely similar to what had been seen in earlier methodologically weaker studies. LVEF decreased by 5%-point and left atrium volume, LV mass, posterior wall thickness and interventricular septum thickness were increased at the end of the AAS cycle (**Figure 6**). The ratio of early (E) to late (A) ventricular filling velocity, or E/A ratio, decreased by 0.45. This hints at decreased LV compliance, i.e., increased LV stiffness. Notably, all parameters returned to baseline at the end of follow-up. However, this does not preclude the possibility that these changes might become permanent with more prolonged AAS use or with repeated cycles that provide too little time for recovery to take place in between. This is especially worrisome as there is considerable evidence that myocardial injury, which may accumulate in years of ongoing AAS use, is a primary cause for sudden cardiac death in AAS users (217).

The small detrimental changes to cardiac structure and systolic and diastolic function are unlikely to lead to clinical complaints, as they principally take place in the subclinical margin even for men frequently performing strenuous exercise. However, they might compound the cardiovascular risk imposed by the other atherogenic effects of AAS, such as dyslipidemia, acting as potential CVD risk modifiers. In hypertensive individuals, LV mass corrected for body surface area adds prognostic value for ischemic heart disease and heart failure in addition to established (SCORE) risk factors (218). The same holds true for global longitudinal strain in nonhypertensive individuals (218). The isovolumic relaxation time is also an independent predictor of heart failure in the general population (219). Consequently, an argument could be made to perceive these AAS-induced cardiac changes as risk modifiers when estimating CVD risk using algorithms such as SCORE2 or PCE, and could aid in ‘grey zone’ risk estimation situations. Nevertheless, it should be acknowledged that firm evidence is lacking.

While it is hard to recommend general echocardiographic screening in this group of patients due to lack of evidence, it can be considered on an individual basis. Detrimental changes to the structure and function of the heart that surface upon echocardiographic examination might also aid in convincing the patient to stop using AAS.

### 3.14 Side effects in women

Although the number of female AAS users is considerably smaller than that of their male counterparts (estimated global lifetime prevalence rate of 1.6% compared with 6.4% for males (2)), female strength athletes use AAS as well. These women tend to perform shorter cycles, favor other AAS types (stanozolol, oxandrolone) and use lower dosages. Even less data than in men describe the effects of AAS use in women. One might extrapolate the effects of supraphysiological androgen levels from men to women with regard to blood pressure, erythrocytosis, lipid profile and cardiac structure. Fertility may be impaired as a result of the suppressive effects of AAS on gonadotropin production, causing disruption of the menstrual cycle. In addition, AAS have virilizing effects, which obviously is not an issue in men but has great clinical significance in women. These effects include dysphonia or deepening of the voice, hirsutism and clitoromegaly.

Dysphonia might occur even at relatively low dosages. Postmenopausal women receiving 50 mg nandrolone decanoate every 4 weeks for a year, superimposed on hormonal replacement therapy (HRT; 2 mg estradiol daily), reported more voice complaints than those receiving HRT only (220). Objectively, there was a loss of high frequencies and a lower mean frequency during speech, as well as increased voice creakiness and instability as assessed by a speech pathologist. Laryngoscopic findings showed no differences between groups with regard to oedema of the vocal cords and laryngitis. However, earlier reports do note oedema and hyperaemia shortly after starting AAS administration, but subsiding afterwards. Corroborating these findings, a dose- and concentration-dependent decrease in average voice pitch was found in women who had undergone a hysterectomy with or without oophorectomy and were randomized to 0, 3, 6.25, 12.5 or 25 mg testosterone enanthate weekly for 24 weeks (221). Importantly, participants did not self-report changes in voice, highlighting that these changes can occur gradually and unnoticed. A case report covering the transition of a female-to-male transgender receiving 200 mg testosterone enanthate bimonthly described a reduction in mean frequency, and lowering and contraction of pitch range within 3–4 months of treatment (222). This suggests that this change might occur relatively rapidly. Importantly, deepening of the voice is thought to be irreversible (88).

Mild hirsutism occurs in around 1 out of 5 women given 150 mg testosterone enanthate every 4 weeks and is reversible after cessation of use (223). Similarly, in postmenopausal women who previously underwent hysterectomy with or without oophorectomy, 12.5 mg and 25 mg testosterone enanthate weekly for 24 weeks led to a small increase in hirsutism (224). Lower dosages up to 6.25 mg weekly did not, suggesting a threshold for developing hirsutism in response to testosterone at a dosage somewhere between 6.25 and 12.5 mg weekly. Based on studies of testosterone treatment in postmenopausal women, the onset of AAS-induced hirsutism appears slow, taking about 4–6 months to become apparent (225).A faster onset can however not be excluded, and the chance of developing hirsutism might be higher with higher dosages of AAS, as seen with illicit use.

Some data about the development of clitoromegaly are available from research in female-to-male transsexual patients. In one study, stretched clitoral length increased from 1.4 cm at baseline to 3 cm after 4 months of receiving 200 mg testosterone cypionate every other week (226). Growth plateaued by 1 year, reaching a mean length of 4.6 cm. Similar results were observed in a study administering 1.000 mg testosterone undecanoate every 3 months (227). Mean clitoral length increased to 4.0 cm after one year of treatment. Yet another study, administering 1.000 mg testosterone undecanoate every 10–14 weeks (titrated to testosterone levels) for 2 years, reported a clitoral length of 2.0, 3.2, 3.3, 3.6, and 3.8 cm at baseline, 3, 6, 12 and 24 months of treatment (228). Clitoral size was unaffected by a lower dosage of 25 mg testosterone enanthate weekly for 24 weeks in hysterectomized women (224). Thus, in contrast with dysphonia and hirsutism, there appears to be a higher threshold of androgenic action required for this side effect to occur.

## 4 Conclusion

AAS are a class of hormones widely used by both amateur and professional athletes. Although they effectively promote the primary goal of increasing muscle strength or size, their use is not without risks. Knowledge of the side effects is exceedingly important for physicians, in particular general practitioners, endocrinologists, sports medicine, and addiction medicine physicians, to recognize associated pathology and provide the right care for this large group of patients. Side effects range from cosmetic issues such as acne vulgaris and clitoromegaly in women, to potentially life-threatening such as cardiovascular disease. Many of the short-term health effects are now well characterized, although the true long-term impact on well-defined clinical endpoints such as cardiovascular morbidity and mortality remains elusive. Large case-control studies are warranted to obtain sufficiently good quality data to provide better insights herein, as it is both virtually infeasible and morally unacceptable to investigate the long-term effects of AAS use in randomized controlled trials.

## Author contributions

The first draft of the manuscript was written by PB. DS and WR commented and contributed to previous versions of the manuscript. All authors read and approved the final manuscript.
